# Ramadan Diurnal Intermittent Fasting Is Associated With Attenuated FTO Gene Expression in Subjects With Overweight and Obesity: A Prospective Cohort Study

**DOI:** 10.3389/fnut.2021.741811

**Published:** 2022-03-17

**Authors:** Mohamed I. Madkour, Lara J. Bou Malhab, Wael M. Abdel-Rahman, Dana N. Abdelrahim, Maha Saber-Ayad, MoezAlIslam E. Faris

**Affiliations:** ^1^Department of Medical Laboratory Sciences, College of Health Sciences, University of Sharjah, Sharjah, United Arab Emirates; ^2^Research Institute for Medical and Health Sciences (RIMHS), University of Sharjah, Sharjah, United Arab Emirates; ^3^Department of Clinical Nutrition and Dietetics, Faculty of Pharmacy, Applied Science Private University, Amman, Jordan; ^4^Department of Clinical Sciences, College of Medicine, University of Sharjah, Sharjah, United Arab Emirates; ^5^Department of Clinical Nutrition and Dietetics, University of Sharjah, Sharjah, United Arab Emirates

**Keywords:** caloric restriction, intermittent fasting, obesity, nutrigenomics, Ramadan, time-restricted eating, gene expression, Middle East

## Abstract

**Aim and Background:**

A growing body of evidence supports the impact of intermittent fasting (IF) on normalizing body weight and that the interaction between body genes and environmental factors shapes human susceptibility to developing obesity. *FTO* gene is one of these genes with metabolic effects related to energy metabolism and body fat deposition. This research examined the changes in *FTO* gene expression upon Ramadan intermittent fasting (RIF) in a group of metabolically healthy subjects with overweight and obesity.

**Methods:**

Sixty-three (63) subjects were recruited, of which 57 (17 males and 40 females, mean age 38.4 ± 11.2 years) subjects with overweight and obesity (BMI = 29.89 ± 5.02 kg/m^2^were recruited and monitored before and at the end of Ramadan month), and 6 healthy subjects with normal BMI (21.4 ± 2.20 kg/m^2^) recruited only to standardize the reference for normal levels of *FTO* gene expression. In the two-time points, anthropometric, biochemical, and dietary assessments were undertaken, and *FTO* gene expression tests were performed using RNA extracted from the whole blood sample.

**Results:**

In contrast to normal BMI subjects, the relative gene expressions in overweight/obese were significantly decreased at the end of Ramadan (−32.30%, 95% CI–0.052 −0.981) in comparison with the pre-fasting state. Significant reductions were found in body weight, BMI, fat mass, body fat percent, hip circumference, LDL, IL-6, TNF-α (*P*<*0.001*), and in waist circumference (*P*<*0.05*), whilst HDL and IL-10 significantly increased (*P*<*0.001*) at the end of Ramadan in comparison with the pre-fasting levels. Binary logistic regression analysis for genetic expressions showed no significant association between high-energy intake, waist circumference, or obesity and *FTO* gene expression.

**Conclusions:**

RIF is associated with the downregulation of the *FTO* gene expression in subjects with obesity, and this may explain, at least in part, its favorable metabolic effects. Hence, RIF presumably may entail a protective impact against body weight gain and its adverse metabolic-related derangements in subjects with obesity.

## Introduction

Obesity is one of the most common prevalent chronic diseases, with its comorbidities and long-term consequent mortality has become a major challenge to global health (1). Obesity is a complicated multifaceted disease that develops from the interaction of cellular, molecular, genetic, metabolic, physiologic, behavioral, cultural, and socioeconomic, influences (2, 3). Notably, cardiovascular disease, diabetes, renal disorders, and neoplasms were the major causes of high body mass index (BMI)-related disability-adjusted life years (DALYs), accounting for 89.3 percent of all high-BMI-related DALYs, with the BMI-related disease burden varying significantly, depending on the Socio-Demographic Index (SDI) (4). Despite the tremendous efforts in the MENA region to combat the problem, yet obesity is still a major health problem due to several factors including the adopted dietary patterns and physical inactivity (5, 6). Genetic predisposing factors represent one of the major contributing factors in the etiopathogenesis of obesity and its consequent complications, with some studies unraveling that high BMI is 25–40% heritable (7). However, to affect body weight, genetic predisposing factors often need to be coupled with environmental and behavioral triggering factors (8, 9).

With the progressive advancement of genome-wide association studies, more than 100 loci have been identified to be associated with obesity and its related traits (10). One of these genetic loci with a strong effect on obesity and related biological functions such as adipogenesis and energy balance regulation, fat mass, and obesity-associated (*FTO*) gene has emerged as one of the influential genes with remarkable impact (11). Recent large-scale analyses found that the obesity-risk allele (rs9939609 A allele) of the *FTO* is associated with increased food intake (12, 13), and previous studies also reported that the *FTO* obesity-risk allele was associated with a reduced response to hunger and satiety after meals in adults and children (14–16).

Nowadays, intermittent fasting (IF) has been looked at as an emerging effective, and costless dietary intervention that helps to promote health and prevent disease and aging (17). Several reports showed the benefits of different styles of IF, including time-restricted eating (TRE, a form of IF that involves confining the eating window to 4–10 h and fasting for the remaining hours of the day) (18), modified fasting regimens allowing 20–25% of energy needs to be consumed on scheduled fasting days, alternate-day fasting. The benefits are well evident for metabolic disorders, as well as cancer, obesity, diabetes, and neurological disorders (17, 19, 20).

Among the widely observed and extensively examined types of IF is the religious form observed during the month of Ramadan (RIF) (21). Ramadan is the ninth month of the lunar calendar, during which healthy adults are mandated to abstain from dawn to sunset, and to refrain from eating and drinking (including water) for a period that extends from 12 to 17 h, depending on the solar season and geographical location (22). This pattern of fasting is associated with dietary (including both food quality and quantity) (23), lifestyles (including sleep quality and quantity) (24) as well as circadian rhythm hormonal changes (25) that may harbor changes in gene expression.

Original research, systematic reviews, and meta-analyses unraveled that observing RIF entails a plethora of beneficial improvements in variable health and metabolic aspects, including reducing the cardiometabolic risk factors such as waist circumference, very low-density-lipoprotein cholesterol, low-density lipoprotein cholesterol (LDL), high-density-lipoprotein cholesterol HDL, total cholesterol (TC), triglycerides (TG), heart rate (HR), systolic blood pressure (SBP), and diastolic blood pressure (DBP) (26, 27). Moreover, RIF is associated reduce body weight (28), body fat content (29) with emphasis on visceral adiposity (30), and ameliorating inflammatory and oxidative stress (31–33), and non-alcoholic fatty liver disease markers in healthy people (34).

With the expansion of the nutrigenomic studies, growing attention has been directed toward examining the effect of different regimens of IF on gene expression of variable genes related to human health and diseases (16, 35). However, there is a paucity of studies tackling the effect of RIF and the associated dietary and lifestyle changes on the expression of specific genes related to human health and disease. Among these, only two studies examined the impact of RIF on the anti-oxidative stress genes (*TFAM, Nrf2, SOD2*) and metabolism-controlling genes (*SIRT1, SIRT3*) (36), the Circadian Locomotor Output Cycles Kaput (*CLOCK)* gene, and other genes related to circadian rhythmicity (37). However, the relationship between RIF and obesity- and body fat-controlling gene expressions is still to be investigated. In the former study, RIF was associated with significant increases in the relative expressions of the antioxidant genes (TFAM, SOD2, and Nrf2) in obese subjects in comparison to counterpart expressions of healthy weight subjects, with percent increments of 90.5, 54.1, and 411.5% for the three genes, respectively. However, the metabolism-controlling gene (SIRT3) showed a highly significant downregulation accompanied by a clear trend for reduction in the SIRT1 gene at the end of Ramadan month, with percent decrements of 61.8 and 10.4%, respectively (36). For the latter, profound changes were reported in the diurnal expression of *CLOCK*, a central component of the circadian molecular clock, during Ramadan compared to the non-fasting month of *Sha'aban* (the month before Ramadan) (37). One study assessed the association of common polymorphisms in the CLOCK and FTO gene polymorphisms (SNPs) (rs1801260 and rs9939609, respectively) with standardized BMI scores, and the impact of dietary and lifestyle modification in school-age children (38). It was found that sex is a potential modifier for the association between the CLOCK polymorphism and BMI z-scores in school-age children (38) and that the FTO SNP, rs9939609, did not significantly modify the effect of the intervention on BMI z-scores at the follow-up or changes of BMI z-scores (38).

Given the proven impact of RIF in lowering body weight (28), body fatness (29), visceral fat content (30), and satiety and eating-controlling hormones (leptin, adiponectin, ghrelin) (25); it becomes rational to examine the relationship between the observance of RIF and the expression of *FTO* gene. Considering the preventive effect of IF, and RIF in particular as a unique diurnal TRE model (39), on the above-mentioned obesity-related indicators, and the principal role of *FTO* in controlling satiety, food intake, body fatness, and obesity risk (11, 13–16); the current work stemmed from the hypothesis that observing RIF will be associated with reduced expression of the *FTO* gene in fasting people with obesity. Therefore, the current work was designed to find out how the observance of RIF by fasting people with obesity will be associated with changes in the genetic expression of *FTO*.

## Methods

### Participant Selection

In total, 63 subjects were recruited, of which 57 (17 males and 40 females, mean age 38.4 ± 11.2 years) subjects with overweight and obesity (BMI = 29.89 ± 5.02 kg/m^2^ (were recruited and monitored before and at the end of Ramadan month, and 6 healthy subjects with normal BMI (21.4 ± 2.20 kg/m^2^) recruited only to standardize the reference for normal levels of *FTO* gene expression. All subjects who visited the University Hospital Sharjah (UHS), UAE, for screening were recruited for this study. All subjects were Arabs from the Arabian Gulf, Iraq, Egypt, Sudan, Tunisia, and the Levant countries (Syria, Jordan, Lebanon, and Palestine). The study protocol was designed and conducted following the Declaration of Helsinki and approved by the UHS Research Ethics Committee (Reference no. REC/16/12/16/002). All enrolled subjects (*n* = 63) were provided with an information sheet describing the research plan, objectives, and requirements of participation. Subjects were recruited using personal communication, social media, and institutional emails. All subjects attended the UHS for screening and investigations and provided signed informed consent to participate in this study. Subjects were men and women who were of either normal weight or overweight/obesity (BMI >25 kg/m^2^) and decided to fast Ramadan and were willing to participate in this study. We have collected basic and sociodemographic data using a self-report questionnaire that covered the medical history and demographic information. The questionnaire was administered in individual face-to-face interviews. A trained research assistant conducted all interviews. The exclusion criteria were a history of metabolic syndrome, diabetes, or cardiovascular disease, taking regular medications for any chronic disease, following a weight-reducing diet, a history of bariatric surgery within the last 6–9 months before commencing Ramadan fasting, and being a pregnant or peri-menopausal woman.

### Study Design

A prospective observational study design was used to investigate the effect of RIF on *FTO* gene expressions along with variable anthropometric, metabolic, and inflammatory markers in subjects with overweight and obesity. Data were collected at baseline (2–7 days before RIF) and after completing 28–30 consecutive days of diurnal RIF. During the fasting month of Ramadan, individuals abstain from all foods and drinks (including water) from dawn to sunset, with the average fasting duration being 15 h per day. Subjects were not requested to follow any dietary or physical activity regimens or recommendations during any stage of this study. All subjects were asked to pursue habitual lifestyle patterns during both fasting and non-fasting hours. According to Islamic laws of fasting, menstruating women are exempted from observing Ramadan fasting during their period; hence, the fasting period for participating women was less than that for men (23–25 vs. 28–30 days).

### Anthropometric Assessment

Anthropometric measurements were taken at two time points (before and at the end of commencement of 28–30 fasting days). Anthropometric measures of body weight, fat mass, and body fat percentage, and fat-free mass were measured using segmental multi-frequency bioelectrical impedance analysis (DSM-BIA; TANITA, MC-980, Tokyo/Japan) before and at the end of the fasting month. The DSM-BIA machine measured the visceral fat rating (from 0 to 100), and this value was converted into a visceral fat surface area by multiplying the obtained value by 10, consistent with the manufacturer's instructions. Height was measured using a fixed stadiometer to the nearest 0.1 cm. BMI was calculated as weight (kg) divided by height in m^2^. Waist and hip circumference were measured to the nearest 0.01 m using a non-stretchable measuring tape (Seca, Hamburg/Germany), and their ratio was calculated accordingly.

### Dietary Intake Assessment

No special dietary recommendations or food regimens were given to the study subjects during any stage of the study, and all the subjects were asked to pursue their habitual dietary patterns during the eating period before and during Ramadan. Dietary intakes were assessed by trained nutritionists using the 24-h recall technique on 3 days (one weekend day and two weekdays) at the two-time points (before and at the end of Ramadan fasting). Printed two-dimensional food models were used to help study subjects approximate the eaten portion sizes. Dietary intakes of energy (calories), macronutrients (carbohydrates, protein, fats, and water), and micronutrients (vitamins and minerals) were estimated using the Food Processor software (version 10.6 ESHA Research, Salem, OR/USA).

### Physical Activity Level

The Dietary Reference Intakes classification for general physical activity level was used to assess subjects' level of physical activity (40). This classification depends on the general physical exercise pattern. Subjects were considered highly active if they performed at least 2 h per day of moderate-intensity physical exercise or 1 h of vigorous exercise in addition to daily living activities. Subjects were considered moderately active if they performed more than 1 h per day of moderate-intensity exercise in addition to daily living activities. Subjects that performed 30 mins to 1 h per day of moderate-intensity physical exercise in addition to daily living activities were considered to have low activity. Finally, subjects who performed daily living activities without other physical exercise were considered sedentary (40).

### Blood Sampling

A sample of 10 ml of blood was collected from subjects at baseline in 3 different tubes including red top (plain) for serum, purple top (EDTA) for plasma and RNA extraction, and gray top (sodium fluoride) for glucose level (before commencing fasting) and at the end of the fasting month. At both time points, blood samples were collected after at least 8 h of fasting. The samples were collected between 11 a.m. and 1 p.m. to eliminate the effect of timing and dietary intake on the measured biochemical parameters and ensure consistency in the duration of fasting for the two-time points. Collected blood samples were divided into two aliquots. One aliquot was centrifuged at 2,500 rpm for 15 min within 1 h of the collection; the serum was aliquoted, coded, and stored at −80°C until it was used for biochemical analysis. The second aliquot was used for RNA extraction, as explained below.

### Biochemical Assay

In this study, we used chemiluminescent immunoassay (CLIA) based on a fully automated clinical chemistry analyzer (Adaltis, Pchem1, Italy) to quantify fasting glucose, total cholesterol (TC), LDL-cholesterol, HDL-cholesterol, and triglycerides (TG) at the two-time points. The pro-and anti-inflammatory cytokines (IL-6 and TNF-α; and IL-10, respectively) were quantified using a multiplex assay (Luminex, Bio-Plex Pro™ Human Cytokine plex Assay).

### Blood Pressure Measurement

Blood pressure was measured before blood sampling using a digital blood pressure monitor (GE, USA), with subjects in an erect, seated position after a 5-min resting period.

### FTO Gene Expression, RNA Extraction, Reverse Transcription, qPCR

RNA was extracted using the column-based, Total RNA Purification kit from Norgen, (Thorold, Canada) and reverse transcribed to cDNA by the QuantiTect Reverse Transcription kit from Qiagen (Hilden, Germany), according to the manufacturer's instructions. cDNA and primer concentrations were optimized to obtain a single amplification peak. qPCR reaction was performed at a volume of 20 μl, including 10 ng of cDNA with 5x HOT FIREPol EvaGreen qPCR Mix Plus (ROX) (Solis Biodyne). The cycling conditions included initial activation of the polymerase for 15 min at 95°C, followed by 45 cycles of 15-s denaturation at 94°C, annealing at 55°C for 30 s followed by extension at 72°C for 30 s. The forward and reverse primers used in the study are presented in Table 1. For each sample, the expression of each gene was normalized to the housekeeping gene ribosomal protein L18 subunit (*RPL*18s); The *RPL*18s was chosen as it showed less variation among *GAPDH* and actin housekeeping genes during the initial optimization; at the same time point. (10ng of cDNA and 0.1 uM of each primer per reaction). Three different negative controls were used at this analysis; control (1) no enzyme was added, control (2) no mRNA was added, and control (3) water was added instead of cDNA (NTC control). Therefore, the minimum amount of cDNA, primers and SYBR green have been used per reaction to obtain the specific signal avoiding false amplification. The relative expression was shown as fold change according to Livak and Schmittgen (41) and was presented as mean and standard deviation as described elsewhere (42). Considering the lack of reference range for *FTO* gene expression, six subjects with normal BMI (21.4 ± 2.20 kg/m^2^) were recruited only to get the normal *FTO* expression levels at the two points (before and after Ramadan fasting). For overweight/obese subjects, at each time point, the *FTO* gene expression was first calculated relative to the housekeeping (*RPL18*) gene, then as a fold-change compared to the gene expression for the normal reference levels obtained from subjects with normal BMI.

**Table 1:** Forward and reverse primers were used in genetic analysis for the *FTO* and housekeeping genes tested.

**Gene**	**Forward primer**	**Reverse primer**
*FTO*	CCAGAACCTGAGGAGAGAATGG	CGATGTCTGTGAGGTCAAACGG
*RPL18*	GATGTCGGATTCTGGAAGTTCC	GGTCAAAGGTGAGGATCTTACCC

### Statistical Analyses

The statistical analyses were done using Statistical analyses were performed using SPSS 24 (IBM, Armonk, NY, USA). and reported based on the Strengthening the Reporting of Observational Studies in Epidemiology (STROBE) guidelines (43). The primary outcome measure was the change in the genetic expression of the *FTO* gene between the two-time points. We estimated that 51 subjects would provide 80% power to detect a significant difference of 5% in genetic expression between baseline (pre-fasting) and post-fasting using a two-tailed paired-samples *t*-test with α = 0.05. With an expected dropout rate of 10%, 56 subjects were planned for enrollment. Tests for normality were included in the model. The variables were expressed as the mean ± standard deviation (SD). Independent sample *t*-test comparing baseline characteristics between males and females. Two-tailed Paired sample *t*-tests were used to compare within-subject changes from baseline (pre-fasting) to post-fasting time points. Binary logistic regression [the odds ratio (OR), 95% confidence interval (CI)] was calculated considering genetic expression as dependent variables, and sex (male vs. female), caloric intake (high, >2,000 Kcal vs. low, <2,000 Kcal), waist circumference as independent variables. We recoded the waist circumference variable as high waist circumference or low waist circumference as per the corresponding sex of the participant. The following criteria were used: High, ≥ 102 cm vs. Normal <102 cm for men and High, ≥ 88 cm vs. Normal <88 cm for women. Linear regression was used to determine the relationship between the change in *FTO* gene expression (dependent variable) before and at the end of Ramadan and biochemical and anthropometric variables (as independent variables). All data were tested at a 5% level of significance (*P* <0.05).

The intra- and inter-assay coefficient variation (CVs) for the tested biomarkers are found through the repository link: https://www.dropbox.com/scl/fi/pghy4x01f6crvy7boihby/Inter-intra-assays-CVs-DNA-15.9.2021.xlsx?dl=0&rlkey=s5j7v8jj5gstdl878fd2k7rgc.

## Results

Fifty-seven (17 males and 40 females, mean age of 38.42 years ± 11.18) overweight/obese subjects (BMI = 29.89 ± 5.02 kg/m^2^) were recruited and monitored before and at the end of fasting the whole month of Ramadan. The majority of subjects were females (about 70%), and most subjects were married (about 83%), university graduates (around 77%), and sedentary (about 91%). About 91% of the study population were from non-Gulf Cooperation Council (GCC) countries (Table 2).

**Table 2:** Subjects' sociodemographic characteristics of the overweight/obese subjects (*n* = 57).

**Characteristic**	***n* (%)**
**Age**, years (Mean ± SD)	38.42 ± 11.18
**Sex**	
Male	40 (70.2)
Female	17 (29.8)
**Nationality**	
UAE and other GCC countries	5 (8.8)
Non-GCC (Palestine, Jordan, Syria, Egypt, Iraq, Tunisia, Sudan)	52 (91.2)
**Marital status**	
Married	47 (82.5)
Single	10 (17.5)
**Educational level**	
Basic education	5 (8.8)
Undergraduate studies	44 (77.2)
Postgraduate studies	8 (14)
**Physical activity**	
Sedentary	52 (91.2)
Low activity	2 (3.5)
Moderately active	3 (5.3)
Highly active	-

*GCC, Gulf Corporation Council; UAE, the United Arab Emirates*.

**Figure 1:**
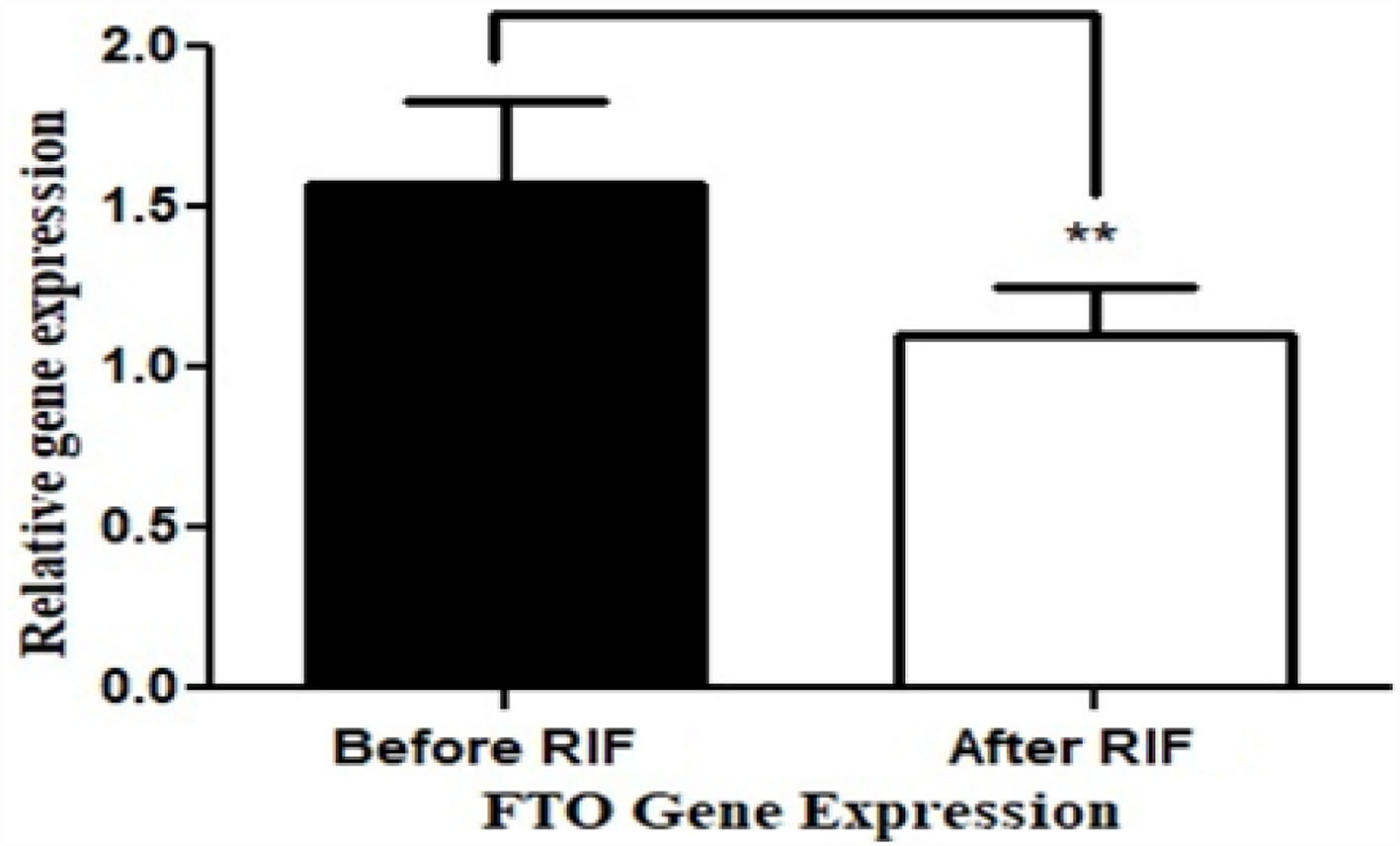
Relative (in comparison with healthy subjects with normal BMI) *FTO* gene expression before and at the end of Ramadan intermittent fasting (RIF) month for the overweight/obese subjects. **P <0.001.

The basic and anthropometric characteristics of the subjects are shown in Table 3. Bodyweight and composition, glucose homeostasis, blood pressure, and inflammatory markers significantly varied between pre-and post-Ramadan fasting, as shown in Tables 4–6. By the end of the Ramadan fasting month, body weight, BMI, fat mass, body fat percent, waist circumference, and hip circumference significantly (*P* <0.05) reduced when compared to pre-fasting levels. LDL-C, IL-6, and TNF-α were significantly reduced as well (*P* <0.05) at the end of the fasting month, while HDL-C and interleukin 10 were significantly increased (*P* <0.05) (Table 5). Changes in dietary intake are shown in Table 4. Significant increases were reported in the dietary intake of total sugars, PUFA, vitamin C, omega-3 fatty acids, lycopene, and vitamin E in comparison with the pre-fasting intakes, while the intake of protein and cholesterol decreases in comparison with the pre-fasting intakes (Table 6). Results of relative genetic expressions in subjects with overweight/obesity showed significant downregulation in the *FTO* gene expression at the end of Ramadan in comparison with the pre-fasting level, with a percent reduction of about−32% (95% CI–0.052 −0.981) (Figure 1).

**Table 3:** Basic anthropometric and cardiac function characteristics of the overweight/obese subjects according to sex variable (*n* = 57).

**Parameter**	**Males (*****n*** **=** **17)**		**Females (*****n*** **=** **40)**		**Significance between males and females**
	**Mean**	**SD**	**Mean**	**SD**	
Weight (kg)	92.52	12.78	78.46	19.44	NS
BMI (kg/m^2^)	29.99	4.20	29.66	6.71	NS
FM (kg)	25.39	8.01	29.15	12.19	NS
BFP (%)	26.86	5.19	35.73	7.17	NS
FFM (kg)	67.13	5.81	49.31	7.56	NS
MM (kg)	63.80	5.54	46.81	7.19	NS
TBW (kg)	47.70	4.30	35.38	5.35	NS
VFA (cm^2^)	114.25	45.79	66.47	38.07	NS
WC (cm)	101.67	11.04	91.53	16.79	NS
HC (cm)	109.64	7.39	111.12	13.34	0.007*
WHR	0.93	0.06	0.82	0.08	NS
SBP (mmHg)	123.47	10.35	124.12	16.13	0.016*
DBP (mmHg)	72.19	8.22	73.00	10.83	NS
Pulse rate (pulse/min)	72.47	8.99	70.00	10.11	NS

*P-value: Independent samples t-test comparing baseline variables between men and women.*

*BMI, Body mass index; BFP, Body fat percent; DBP, Diastolic blood pressure; FM, Fat mass; FFM, Fat-free mass; HC, Hip circumference; MM, Muscle mass; SBP, Systolic blood pressure; TBW, Total body water; VFA, Visceral fat area measured by DSM-BIA; WC, Waist circumference; WHR, Waist: hip ratio.*

*P <0.05, significant difference.

**Table 4:** Changes in anthropometric and cardiac functions measured before and at the end of Ramadan for the overweight/obese subjects (*n* = 57).

**Parameter**	**Before Ramadan(T1)**		**At the endof Ramadan(T2)**		**Significance as compared to baseline**
	**Mean**	**SD**	**Mean**	**SD**	
Weight (kg)	88.32	16.24	86.73	15.74	0.001**
BMI (kg/m^2^)	29.89	5.02	29.40	4.94	0.001**
FM (kg)	26.51	9.49	25.25	9.40	0.001**
BFP (%)	29.51	7.09	28.58	7.34	0.001**
FFM (kg)	61.81	10.37	60.94	10.99	NS
MM (kg)	58.73	9.88	58.41	9.57	NS
TBW (kg)	44.02	7.31	43.84	7.01	NS
VFA (cm^2^)	100.00	48.59	95.72	45.11	NS
WC (cm)	98.64	13.69	97.23	13.03	0.030*
HC (cm)	110.08	9.46	108.55	8.87	0.001**
WHR	0.89	0.08	0.89	0.08	NS
SBP (mmHg)	123.66	12.21	125.64	12.84	NS
DBP (mmHg)	72.43	8.99	73.53	10.26	NS
Pulse Rate (pulse/min)	71.73	9.32	72.13	9.25	NS

*Paired t-test comparing the end of RIF (T2) with pre-fasting baseline (T1).*

*BMI, Body mass index; BFP, Body fat percent; FM, Fat mass; BFP, Body fat percent; FFM, Fat free mass; MM, Muscle mass; TBW, Total body water; VFA, Visceral fat area measured by DSM BIA; WC, Waist circumference; HC, Hip circumference; WHR, Waist: hip ratio; SBP, Systolic blood pressure; DBP, Diastolic blood pressure.*

*P <0.05, significant difference.

**P <0.001, highly significant difference.

**Table 5:** Changes in glucose homeostasis and inflammatory markers before and at the end of Ramadan for the overweight/obese subjects (*n* = 57).

**Parameter**	**Before Ramadan(T1)**		**At the endof Ramadan(T2)**		**Significance as compared to baseline**
	**Mean**	**SD**	**Mean**	**SD**	
FBG (mg/dl)	99.91	20.54	105.70	24.28	NS
TC (mg/dl)	173.95	39.07	175.88	35.81	NS
HDL-C (mg/dl)	45.65	6.77	58.35	11.70	0.001**
TG (mg/dl)	93.63	53.83	97.42	44.10	NS
LDL-C (mg/dl)	109.58	32.55	98.02	33.91	0.001**
Interleukin-6 (pg/dl)	29.86	16.75	18.21	1.29	0.001**
TNF-α (pg/dl)	28.17	4.40	21.24	1.49	0.001**
Interleukin-10 (pg/dl)	18.25	0.71	19.44	1.21	0.001**

*Paired t-test comparing the end of RIF (T2) with pre-fasting baseline (T1).*

**P <0.001, highly significant difference.

**Table 6:** Changes in the dietary intake before and at the end of Ramadan for the overweight/obese subjects (*n* = 57).

**Nutrient**	**Before Ramadan(T1)**		**At the endof Ramadan(T2)**		**Significance as compared to baseline**
	**Mean**	**SD**	**Mean**	**SD**	
Energy (kcal/d)	2,123.08	754.20	2,150.43	847.11	NS
Fat calories (kcal/day)	694.50	396.38	687.07	366.23	NS
Protein (g/d)	108.35	36.61	89.93	39.25	0.002*
Total carbohydrates (g/d)	253.66	94.84	282.74	122.88	NS
Total sugars (g/d)	65.90	31.33	107.73	53.71	0.001**
Total fats (g/d)	77.32	44.08	76.76	41.10	NS
Saturated Fat	23.49	12.55	22.66	12.20	NS
Total water (ml/day)	1,397.02	690.34	1,518.62	808.16	NS
MUFA (g/d)	20.23	11.24	23.05	13.78	NS
PUFA (g/d)	10.47	8.09	16.32	16.81	0.016*
*Trans* fat (mg/d)	0.50	0.81	0.47	1.21	NS
Cholesterol (mg/d)	394.85	176.89	272.56	181.90	0.001**
Vitamin C (mg/d)	73.50	50.63	97.43	66.71	0.006*
α-Carotene (μg/d)	12.61	22.82	16.24	33.30	NS
β-Carotene (μg/d)	393.88	657.97	576.24	938.61	NS
Omega 3 (mg/d)	0.66	0.56	1.82	2.29	0.001**
Omega 6 (mg/d)	7.84	7.08	10.06	10.70	NS
Lycopene (μg/d)	1,484.37	3,493.41	4,234.36	9,700.64	0.048*
Vitamin E (mg/d)	5.75	3.57	8.49	9.44	0.042*
Selenium (μg/d)	85.84	48.50	70.98	47.22	NS

*Paired t-test comparing the variables at the end of RIF (T2) with pre-fasting baseline (T1).*

*MUFA, Monounsaturated fat; PUFA, Polyunsaturated fat.*

*P <0.05, significant difference.

**P <0.001, highly significant difference.

Binary logistic regression analysis for genetic expressions showed no significant (*P* > 0.05) association between high-energy intake (≥2,000 kcal vs. <2,000 kcal), waist circumference (High, ≥ 102 cm vs. Normal <102 cm for men and High, ≥ 88 cm vs. Normal <88 cm for women), obesity (BMI ≥ 30 vs. BMI <30) and gene expressions of *FTO* gene (Supplementary Table 1). Linear regression analysis showed a significant, but weak, positive association between the hip circumference and the *FTO* gene expression at the end of Ramadan fasting days (Supplementary Table 2).

## Discussion

The current study provides the first evidence of a link between RIF and the *FTO* gene expression in a cohort of overweight/obese subjects who observed Ramadan fasting (28 days for an average of 15 h/day). There was an association between reduced *FTO* expression and favorable effects, as demonstrated by suppression of pro-inflammatory markers and improvement of the lipid profile. Above all, such an association was also accompanied by a reduction of BMI and waist/hip ratio denoting that the effect of RIF may be explained, at least in part, by its link to the *FTO* expression. Several human studies showed beneficial effects of IF (17, 19, 21, 44, 45). Recently, a growing body of evidence suggests substantial health implications for the religious form of IF, with the Ramadan model as one of the most extensively studied forms with variable anthropometric, metabolic, and inflammatory impacts (25–29, 31, 32). The main distinctive features of RIF in comparison to other patterns of IF models are presented in the fact that RIF involves diurnal, dawn to sunset, IF for 29–30 consecutive days with complete abstinence from food and drink, including water. Other models of IF include modified fasting regimens (involves consumption of 20–25% of energy needs on scheduled fasting days such as 5:2 diet), TRE (allows *ad libitum* nutrient and energy intake within specific time frames, and inducing regular, extended fasting intervals, mostly nocturnal fasting), and alternate-day fasting (allows alternating fasting days with eating days) (45).

In the current study, RIF reduced body weight, BMI, body fat percent, waist circumference; RIF also resulted in a reduction of LDL, with increased HDL. This is in support of the previous reports on the favorable effect of RIF on the cardiometabolic risk factor profile. In our recent meta-analysis, we demonstrated the favorable effect of IF on reducing total cholesterol, LDL, triglyceride levels as well as diastolic blood pressure and heart rate (26). The study included subgroup analysis of age, sex, and duration of fasting (as confounding factors) and the significant favorable effect of RIF was constantly evident in all subgroups. Concordant with our findings in the current and previous studies, Mindikoglu et al. showed that RIF resulted in a significant reduction of BMI, waist circumference, and improvement in blood pressure, with an anti-cancer, anti-diabetes, and anti-aging serum proteome response, providing another dimension for the benefits of IF that is likely to be promoted by cytokine modulation (46).

The observed changes in the total energy and dietary intakes from different macro and micronutrients are repeatedly shown in several studies (30, 32), and consistent with the recent work that compared dietary intakes from different food groups and macronutrients in a comparative study using the year-round dietary intakes (23).

*FTO* expression is different in underfeeding and fasting conditions and displays tissue-specific differences in mouse models of obesity, but it is not known whether these differences are the cause or the consequence of obesity (47). *FTO* mRNA expression in mice and humans is broadly distributed in many organs, with notably high levels of expression in the brain and hypothalamus, which regulate energy balance and hunger (48, 49). Previous studies showed that tissue-specific genes may be expressed in a wider variety of tissues. When transcriptomics analysis of peripheral blood mononuclear cells (PBMCs) was compared to that of liver, kidney, stomach, spleen, prostate, lung, heart, colon, and brain, more than 80% of shared differentially expressed genes (50). Also, other reports suggested that PBMCs can represent a surrogate indicator in dietary investigations to identify differentially expressed genes in population studies (51, 52). In mice, the expression of FTO may be influenced by their dietary condition (53). When mice are fasting, there is a strong stimulus to eat, and their hypothalamus *FTO* mRNA expression is significantly reduced compared to their fed counterparts. Supplementation with the anti-hunger hormone leptin does not reverse this effect, which suggests that the reduced hypothalamic FTO expression observed during fasting is independent of leptin levels (54). These findings show that FTO is downregulated during fasting and increased during feeding and that a decrease in FTO expression or activity might be a signal that encourages overeating and obesity. Noteworthy, the results in rats are different; possibly because of inconsistency in the conditions of different studies and the different sensitivity to starvation among different species. Murine FTO gene expression was shown to be downregulated under fasting conditions, suggesting that obese mouse models mimic the fasted state, possibly contributing to their over-eating (47).

Interestingly, *FTO* was highly expressed in the cerebellum, salivary gland, and kidney of adult pigs, whereas it was not detected in blood (55). The latter study showed *FTO* was positively associated with energy intake in the pancreas, and with age in the muscle, adding to the multiple factors that affect FTO expression. Such variation can be explained by different metabolic and secretion activities of different tissues at different age groups. Moreover, as previously described with leptin, diurnal variation may also affect the level of expression of *FTO* (56). The link of *FTO* expression to fasting and obesity is not yet fully elucidated in humans, where more factors may interplay to determine this effect. Such factors include the effect of food predilection, dietary patterns, and complexity of gut-brain networking including leptin, ghrelin, among other key players (57). Our current study highlights the reduction of *FTO* in overweight/obese subjects as a consequence of observing diurnal IF for four consecutive weeks.

Furthermore, the current study showed a reduction in both pro-inflammatory cytokines IL6, TNF-α. Concordantly, in a study by Faris et al. significant reductions in IL-6, IL-1β, and TNF-α were reported in fasting subjects during Ramadan of both sexes, when compared to basal pre-fasting values obtained 1 week before Ramadan (32). Furthermore, this finding is consistent with the results of a meta-analysis and original research showing that RIF is associated with significant reductions in serum proinflammatory cytokines (IL-6, IL-1β, and TNF-α) and *hs*-CRP, and the oxidative stress marker malondialdehyde and urinary 15-f(2t)-isoprostane (32, 33). The current findings on the significant reductions in lipid profile components (TC, LDL, and TG) and increased HDL are consistent with the systematic reviews and meta-analyses showing that RIF is associated with such improvements in the cardiometabolic risk factors (26, 27). As shown by Faris and colleagues (30), these reductions in the proinflammatory cytokines and other inflammatory adipokines were reported to be associated with significant reductions in visceral adiposity in obese subjects who observed the 4-week dawn to sunset IF of Ramadan. Experimentally, fasting reduced TNF-α in visceral white adipose tissue, IL-1β in subcutaneous tissue, as well as insulin and leptin in the plasma in stressed rats (58).

The current study showed that RIF increased IL-10, which is consistent with a previous study by Faris et al. among obese subjects observing RIF when compared with the pre-fasting levels (30). IL-10 has a strong immune-modulation activity (59). It is thought of as an anti-inflammatory cytokine that can suppress cytokine production from macrophages and the function of neutrophils (60, 61) but can activate CD8+ T cells and natural killer (NK) cells for anti-viral immunity, denoting its dual role in immunity (62, 63). Intriguingly, the IL-10 signaling pathway was one of the top Differentially Expressed Genes (DEGs) in COVID-19 infected normal epithelium vs. mock-infected cells (64) and could be, along with the reduction in other metabolic and inflammatory risk factors, involved in the plausible protective effect of Ramadan fasting against the COVID-19 infection (65).

*FTO* expression did not correlate with high-energy intake, waist circumference, or obesity as shown by the binary logistic regression analysis performed. These findings denote that RIF exerts its beneficial effects independently from the dietary and anthropometric factors, through different pathways that may or may not involve weight reduction and lower energy intake. Such dissociation between the beneficial effect of IF and caloric restriction is supported by previous work on rodents (66) who found that IF has beneficial effects in experimental mice reported on glucose regulation and neuronal resistance to injury that are independent of caloric intake. Several proteins interact with the *FTO*; the most significant of which is Melanocortin receptor 4 (MCR4) that is co-expressed with the *FTO* in some species. The MCR4 is involved in energy balance as well as somatic growth (67).

Pharmacologic treatments cannot reset the circadian clock rhythm; thus, there is an urgent need for an effective intervention to reset the circadian clock and prevent metabolic syndrome and metabolic syndrome-induced cancers (68, 69). However, IF practiced exclusively during human activity hours can reset the circadian rhythm. Therefore, resetting the disrupted circadian clock in humans by consecutive daily IF could provide a primary strategy to improve metabolic syndrome and reduce the incidence of metabolic syndrome-induced cancer (68, 69). RIF upregulated several key regulatory proteins that play a key role in tumor suppression, DNA repair, insulin signaling, glucose, and lipid metabolism, circadian clock, cytoskeletal remodeling, immune system, and cognitive function (70).

Nonetheless, our results indicate a lack of association between *FTO* gene expression and caloric intake by the fasting people. This notion may appear inconsistent with the evident association between intakes of calories, carbohydrates, and fats with the *FTO* genotype (67), given some studies that showed a correlation of the *FTO* risk allele and high *FTO* gene expression (68, 69). Moreover, it has been reported that the *FTO* genotype may influence dietary macronutrient intakes, body weight, energy balance, appetite, and hormone secretion (70, 71). The SNPs of the *FTO* gene are likely associated with food intake and obesity through modifying the expression of other genes (72). Until now, there is no strong evidence for the association of *FTO* A risk allele and level of gene expression. There is a recognized association of the A risk allele of *FTO rs9939609* and overweight worldwide (48, 71) in several Arab populations (72–75). In this study, we did not investigate the subjects' genotypes, as the study group is not from the same ethnicity. In our previous study on the Emirati population, the rs9939609 AA genotype was significantly associated with higher BMI; in females, but not in males (76). In another study by our group, subjects with *rs9939609* AA genotype showed significantly higher fasting glucose compared to other genotypes, with a trend of higher insulin levels and HOMA2-IR (77). We recently correlated the *FTO* genotypes, as well as FGF21 genotypes to dietary patterns in the Emirati population (78). Whether the outcome of a caloric intervention is affected by *FTO* rs 9939609 A risk allele is weakly evident (79).

A few limitations should be considered when interpreting the findings of the current work. First, causality cannot be inferred, as the design is observational prospective in nature. Hence, undetected confounding factors could be involved in the downregulation or upregulation of the tested *FTO* gene upon RIF. Changes in circadian rhythm and sleep patterns that have been reported to affect the expression of some genes (37) are among the factors that may be implicated in changing *FTO* gene expression. Tissue- and age-specific variations of *FTO* expression also add to the complexity of the interpretation of its expression in the blood. Although the practice of physical exercise did not change during Ramadan month in comparison with the pre-fasting stage, still this factor may be of paramount effect, and objective measurements have to be applied in measuring physical exercise levels in the forthcoming studies.

## Conclusions

RIF is linked to the downregulation of *FTO* gene expression in subjects with obesity, which might explain, at least in part, its beneficial metabolic benefits. Consequently, RIF may have a preventive effect against body weight increase and associated negative metabolic-related derangements in overweight/obese people, possibly through modulation of *FTO* gene expression.

## Data Availability Statement

The original contributions presented in the study are included in the article/Supplementary Material, further inquiries can be directed to the corresponding author/s.

## Ethics Statement

The studies involving human participants were reviewed and approved by Research Ethics Committee, University of Sharjah. The patients/participants provided their written informed consent to participate in this study.

## Author Contributions

MF, MS-A, and MM: conceptualization and validation. MF, MM, and LM: data curation and methodology. DA: formal analysis. MF, MS-A, and WA-R: funding acquisition, supervision, and project administration. MF, MS-A, MM, and LM: investigation. MF and WA-R: resources. MF and WA-R: software. LM, MM, and DA: visualization. MF and MS-A: writing—original draft. MF, MS-A, and WA-R: writing—review and editing. All authors contributed to the article and approved the submitted version.

## Funding

Vice-Chancellor Research has supported this work and Graduate Studies Office/the University of Sharjah, Grant No. (VCRG/R1061/2016). MS-A was funded by the University of Sharjah targeted Grant (1801090141-P) and the MBRU-Al-Mahmeed Research Award 2019, WA-R was funded by the University of Sharjah Grant 16010501020-P.

## Conflict of Interest

The authors declare that the research was conducted in the absence of any commercial or financial relationships that could be construed as a potential conflict of interest.

## Publisher's Note

All claims expressed in this article are solely those of the authors and do not necessarily represent those of their affiliated organizations, or those of the publisher, the editors and the reviewers. Any product that may be evaluated in this article, or claim that may be made by its manufacturer, is not guaranteed or endorsed by the publisher.
