# Supplementary material for: Ramadan Diurnal Intermittent Fasting Is Associated With Attenuated FTO Gene Expression in Subjects With Overweight and Obesity: A Prospective Cohort Study
Source: Front Nutr. 2022 Mar 17;8:741811. doi: 10.3389/fnut.2021.741811 (PMC8968860; doi:10.3389/fnut.2021.741811)
Supplement: Supplementary file 1 [file Data_Sheet_1.docx]

**Suppl. Table 1**. Correlations between relative *FTO* gene expression with biochemical and anthropometric factors before and at the end of Ramadan fasting month for the overweight/obese subjects (n = 57).

| **Parameter** | **Before Ramadan**  **(T1)** | | **Significance** | **At the end of Ramadan**  **(T2)** | | **Significance** |
| --- | --- | --- | --- | --- | --- | --- |
|  | *r* - value | *P*-value |  | *r* - value | *P*-value |  |
| FM (kg) | -0.063 | 0.654 | NS | 0.263 | 0.057 | NS |
| BFP (%) | -0.060 | 0.667 | NS | 0.136 | 0.332 | NS |
| FFM (kg) | 0.106 | 0.449 | NS | 0.167 | 0.232 | **NS** |
| MM (kg) | 0.108 | 0.442 | NS | 0.186 | 0.181 | NS |
| TBW (kg) | 0.146 | 0.297 | NS | 0.105 | 0.456 | NS |
| VFA (cm^2^) | 0.011 | 0.939 | NS | 0.010 | 0.943 | NS |
| WC (cm) | -0.102 | 0.468 | NS | 0.095 | 0.501 | NS |
| HC (cm) | -0.039 | 0.779 | NS | 0.267 | 0.054 | NS |
| SBP (mmHg) | 0.117 | 0.403 | NS | -0.057 | 0.684 | NS |
| DBP (mmHg) | -0.044 | 0.755 | NS | -0.140 | 0.316 | NS |
| HR (Pulse/min) | 0.204 | 0.144 | NS | 0.235 | 0.090 | NS |
| FG (mg/dl) | -0.235 | **0.017** | * | 0.010 | 0.944 | NS |
| TC (mg/dl) | -0.106 | 0.450 | NS | -0.126 | 0.370 | NS |
| HDL (mg/dl) | -0.089 | 0.528 | NS | 0.197 | 0.157 | NS |
| TGs (mg/dl) | -0.147 | 0.292 | NS | -0.179 | 0.200 | NS |
| LDL (mg/dl) | -0.062 | 0.660 | NS | -0.154 | 0.270 | NS |
| IL-6 (pg/dl) | -0.034 | 0.807 | NS | 0.003 | 0.985 | NS |
| IL-10 (pg/dl) | 0.129 | 0.357 | NS | -0.020 | 0.889 | NS |
| TNF-α (pg/dl) | -0.266 | 0.054 | NS | -0.144 | 0.303 | NS |

Pearson correlation test between FTO gene with biochemical, anthropometric, and dietary risk factors at pre-fasting baseline (T1) and the end of RIF (T2), after being adjusted for age, sex, BMI, and calorie intake.

* *P* < 0.05, significant difference.

BFP, Body fat percent; BFP, Body fat percent; DBP, Diastolic blood pressure; FFM, Fat-free mass; FG, Fasting glucose;

FM, Fat mass; HC, Hip circumference; HDL, High-density lipoprotein cholesterol; HR, Heart Rate; IL-10, Interleukin-10IL-6, Interleukin-6; LDL, Low-density lipoprotein cholesterol; MM, Muscle mass; SBP, Systolic blood pressure;

TBW, total body water; TC, total cholesterol; TG, Triglycerides; TNF-α, Tumor-necrosis factor-alpha; VFA, Visceral fat area measured by DSM-BIA; WC, Waist circumference; WHR, Waist: hip ratio.

**Suppl. Table 2.** Binary logistic regression analysis for genetic expressions between

anthropometric and dietary variables at the end of Ramadan for the overweight/obese subjects

| **Variable** | **Relative *FTO* gene expression at the end of Ramadan fasting** |
| --- | --- |
|  | **Mean ± SD**  **or correlation coefficient (*r*) (95% CI)** |
| Males *vs.* Females | |
| Males (n=17) | 1.20 ± 1.23 |
| Females (n=40) | 0.88 ± 0.73 |
| High caloric *vs.* low caloric diet | |
| ≥ 2000 kcal (n= 31) | 0.927 (0.571 – 1.507) |
| < 2000 kcal (n=26) |  |
| High WC *vs.* Low WC | |
| High WC ** (n=29) | 0.612 (0.322 – 1.164) |
| Low WC (n=28) |  |
| Obese *vs.* non-obese | |
| Obese (BMI ≥30) (n=22) | 0.864 (0.534 – 1.399) |
| Non-obese (BMI <30) (n=35) |  |

*Significant difference at *P*<0.05.

**High waist circumference (WC) considered high as ≥ 102 cm for male as and ≥ 88 cm female.
